# Type 1 diabetes genetic risk score discriminates between monogenic and Type 1 diabetes in children diagnosed at the age of <5 years in the Iranian population

**DOI:** 10.1111/dme.14071

**Published:** 2019-07-25

**Authors:** H. Yaghootkar, F. Abbasi, N. Ghaemi, A. Rabbani, M. N. Wakeling, P. Eshraghi, S. Enayati, S. Vakili, S. Heidari, K. Patel, F. Sayarifard, S. Borhan‐Dayani, T. J. McDonald, S. Ellard, A. T. Hattersley, M. M. Amoli, R. Vakili, K. Colclough

**Affiliations:** ^1^ Genetics of Complex Traits University of Exeter Medical School, Royal Devon & Exeter Hospital Exeter UK; ^2^ Growth and Development Research Centre Tehran University of Medical Sciences Tehran Iran; ^3^ Department of Paediatric Disease Faulty of Medicine, Mashhad University of Medical Sciences Mashhad Iran; ^4^ Institute of Biomedical and Clinical Science University of Exeter Medical School Exeter UK; ^5^ Metabolic Disorders Research Centre Endocrinology and Metabolism Molecular-Cellular Sciences Institute Tehran University of Medical Sciences Tehran Iran; ^6^ Medical Genetics Research Centre Mashhad University of Medical Sciences Mashhad Iran; ^7^ Departments of Clinical Biochemistry Royal Devon and Exeter NHS Foundation Trust Exeter UK; ^8^ Departments of Molecular Genetics Royal Devon and Exeter NHS Foundation Trust Exeter UK

## Abstract

**Aim:**

To examine the extent to which discriminatory testing using antibodies and Type 1 diabetes genetic risk score, validated in European populations, is applicable in a non‐European population.

**Methods:**

We recruited 127 unrelated children with diabetes diagnosed between 9 months and 5 years from two centres in Iran. All children underwent targeted next‐generation sequencing of 35 monogenic diabetes genes. We measured three islet autoantibodies (islet antigen 2, glutamic acid decarboxylase and zinc transporter 8) and generated a Type 1 diabetes genetic risk score in all children.

**Results:**

We identified six children with monogenic diabetes, including four novel mutations: homozygous mutations in *WFS1* (*n*=3), *SLC19A2* and *SLC29A3*, and a heterozygous mutation in *GCK*. All clinical features were similar in children with monogenic diabetes (*n*=6) and in the rest of the cohort (*n*=121). The Type 1 diabetes genetic risk score discriminated children with monogenic from Type 1 diabetes [area under the receiver‐operating characteristic curve 0.90 (95% CI 0.83–0.97)]. All children with monogenic diabetes were autoantibody‐negative. In children with no mutation, 59 were positive to glutamic acid decarboxylase, 39 to islet antigen 2 and 31 to zinc transporter 8. Measuring zinc transporter 8 increased the number of autoantibody‐positive individuals by eight.

**Conclusions:**

The present study provides the first evidence that Type 1 diabetes genetic risk score can be used to distinguish monogenic from Type 1 diabetes in an Iranian population with a large number of consanguineous unions. This test can be used to identify children with a higher probability of having monogenic diabetes who could then undergo genetic testing. Identification of these individuals would reduce the cost of treatment and improve the management of their clinical course.

## What's new?

- Studies in white European populations have recently shown that a genetic risk score for Type 1 diabetes has a high ability to discriminate between Type 1 diabetes and monogenic diabetes.
- The diagnostic utility of this genetic risk score in non‐European populations is unknown.
- This study provides the first evidence that the Type 1 diabetes genetic risk score discriminates children with monogenic diabetes from those with Type 1 diabetes in the Iranian population with a large number of consanguineous unions.
- The Type 1 diabetes genetic risk score can be used to improve the selection of non‐European children for monogenic diabetes testing, resulting in the correct diagnosis, improving their clinical management and providing families with recurrence risk information.

**What's new?**

- Studies in white European populations have recently shown that a genetic risk score for Type 1 diabetes has a high ability to discriminate between Type 1 diabetes and monogenic diabetes.
- The diagnostic utility of this genetic risk score in non‐European populations is unknown.
- This study provides the first evidence that the Type 1 diabetes genetic risk score discriminates children with monogenic diabetes from those with Type 1 diabetes in the Iranian population with a large number of consanguineous unions.
- The Type 1 diabetes genetic risk score can be used to improve the selection of non‐European children for monogenic diabetes testing, resulting in the correct diagnosis, improving their clinical management and providing families with recurrence risk information.

## Introduction

The accurate diagnosis of diabetes subtypes is challenging, especially in young children in whom monogenic diabetes is often misdiagnosed as Type 1 diabetes 1, 2, 3, 4, 5. The correct diagnosis is crucial because the best management for each subtype is different. People with Type 1 diabetes require lifelong insulin treatment, while those with particular monogenic diabetes subtypes such as *GCK*,* HNF1A* and *HNF4A* maturity‐onset diabetes of young (MODY) can be treated without insulin 6, 7. Misdiagnosis of monogenic diabetes as Type 1 diabetes can result in unnecessary insulin treatment, causing suboptimal glucose control, higher management costs and avoidable side effects. Furthermore, correct diagnosis improves clinical care by guiding anticipation of the development of related features and enabling testing for at‐risk family members 7, 8, 9.

The likelihood of diagnosing monogenic diabetes in paediatric cohorts can be improved by the use of biomarkers for Type 1 diabetes. Combined islet autoantibody testing against glutamic acid decarboxylase (GAD), islet antigen 2 (IA2) and zinc transporter 8 (ZnT8) can discriminate between autoimmune Type 1 diabetes and monogenic diabetes with a high degree of sensitivity and specificity 10, 11, 12, 13. The Type 1 diabetes genetic risk score is a more recent discriminative tool for Type 1 diabetes that is calculated based on the number of risk alleles (weighted by their effect on risk of Type 1 diabetes) each individual carries 14, 15. Studies of white European populations with low rates of consanguinity (1–4% of marriages 16) have shown that the Type 1 diabetes genetic risk score has a high ability to discriminate between Type 1 diabetes and monogenic diabetes, enabling the exclusion of people with probable Type 1 diabetes from inappropriate genetic testing 12, 17. Discriminatory testing using antibodies and Type 1 diabetes genetic risk score has been developed and validated in European populations only, and the extent to which these tests improve the accurate diagnosis of diabetes subtypes in other populations is not known.

By testing autoantibodies, using a Type 1 diabetes genetic risk score and sequencing of all known monogenic diabetes genes in an unselected paediatric diabetes cohort, we aimed to determine whether triple antibody testing (GAD, IA2 and ZnT8) and the Type 1 diabetes genetic risk score could distinguish monogenic diabetes from Type 1 diabetes in the Iranian population where >30% of marriages are consanguineous 18, 19, 20. We also report for the first time the frequency of islet autoantibodies and prevalence of monogenic subtypes in Iranian children with diabetes using a genetic test for all subtypes of monogenic diabetes.

## Participants and methods

### Study participants

We recruited 127 unrelated children with diabetes diagnosed between the ages of 9 months and 5 years from two centres in Iran [Imam Reza Hospital, Mashhad, Iran and the Division of Endocrinology and Metabolism in the Department of Paediatrics at the Children's Medical Centre in Tehran, Iran (Table 1)]. Clinical information was supplied by the referring clinicians. Informed consent was obtained from parents on behalf of their children. Peripheral blood samples were collected from affected children and their parents at the time of referral and used to measure islet autoantibodies and perform genetic testing.

**Table 1:** Clinical characteristics of the cohort

	All	Monogenic diabetes	Type 1 diabetes	*P* for difference
Number of children (boys, girls)	127 (63, 64)	6 (2, 4)	121 (61, 60)	0.70
Age at diagnosis, years (IQR)	3 (2–4)	3.2 (2–4.4)	3.3 (1.9–4.1)	0.84
Significant regions of homozygosity, *n* (%)	63 (49.6)	5 (83.3)	58 (47.9)	0.11
Last HbA_1c_, mmol/mol (IQR)	65.0 (56.3–79.2)	66.1 (57.4–103.3)	65.0 (56.3–79.2)	0.76
Last HbA_1c_, % (IQR)	8.1 (7.3–9.4)	8.2 (7.4–11.6)	8.1 (7.3–9.4)	0.76
GAD‐positive, *n* (%)	59 (47.2)	0 (0)	59 (48.8)	0.03
IA2‐positive, *n* (%)	39 (31.2)	0 (0)	39 (32.2)	0.18
ZnT8‐positive, *n* (%)	31 (24.8)	0 (0)	31 (25.6)	0.34
Positive for at least one antibody, *n* (%)	84 (67.2)	0 (0)	84 (69.4)	0.001
Positive for two antibodies, *n* (%)	21 (16.8)	0 (0)	21 (17.4)	0.58
Positive for three antibodies, *n* (%)	12 (9.6)	0 (0)	12 (9.9)	1
Insulin treatment at diagnosis, *n* (%)	124 (97.6)	4 (66.7)	120 (99.2)	0.006
Insulin treatment at recruitment, *n* (%)	125 (98.4)	4 (66.7)	119 (98.4)	0.006
Syndromic features, *n* (%)	38 (30)	3 (50)	35 (28.9)	0.36
Duration of diabetes, days	589 (53–1689)	710 (37–1746)	589 (61–1684)	0.51
Parent affected with diabetes, *n* (%)	12 (9.4)	1 (16.7)	11 (9.1)	0.51
Type 1 diabetes genetic risk score (IQR)	10.8 (9.5–11.6)	8.4 (8–8.8)	10.8 (9.7–11.6)	0.005

GAD, glutamic acid decarboxylase; IA2, islet antigen 2; IQR, interquartile range; ZnT8, zinc transporter 8.

### Cohort characteristics

Our cohort of 127 children included 64 girls and 63 boys; of these 41 children came from consanguineous families (32.2%; Table 1). The median [interquartile range (IQR)] age at diagnosis was 3 (2–4) years. A total of 125 children (98%) were on insulin treatment at the time of study recruitment. Two children (2%) were non‐insulin‐treated, including one child who was receiving oral agents. The median (IQR) last HbA_1c_ value was 65.0 (56.3–79.2) mmol/mol [8.1 (7.3–9.4)%] for 98 children with data available.

### Genetic testing

DNA was extracted, using standard methods, at the Exeter Molecular Genetics Laboratory (Exeter, UK). All children underwent targeted next‐generation sequencing of 35 known monogenic diabetes genes (Table S1) as previously described 14, 21. All putative mutations were confirmed by Sanger sequencing or digital droplet PCR (primers available on request) in the probands and both parents.

Variants were classified according to the American College of Medical Genetics and Genomics and the Association for Molecular Pathology standards and guidelines for the interpretation of sequence variants 22. We checked the frequencies of the identified variants in GnomAD [>120 000 individuals (http://gnomad.broadinstitute.org)] and in human variant and mutation databases, such as ClinVar and Human Gene Mutation Database, as well as in the literature via PubMed and Google searches. The *in silico* tools SIFT, PolyPhen‐2 and Align GVGD were used to assess the pathogenicity of missense variant effects, and the prediction of variant effect on mRNA splicing was made using SpliceSiteFinder‐like, MaxEntScan, GeneSplice, NNSPLICE and Human Splicing Finder. All *in silico* programs were accessed through the ALAMUT Visual software version 2.7.1 (Interactive Biosoftware, Rouen, France). Conservation of amino acids and nucleotides across multiple species was performed using the University of California Santa Cruz genome browser (http://genome.ucsc.edu).

### Antibody testing

The GAD, IA2 and ZnT8 antibody testing was performed by the Exeter Academic Department of Blood Sciences at the Royal Devon and Exeter Hospital (Exeter, UK). We used commercially available ELISA assays (RSR, Cardiff, UK) on the Dynex DS2 ELISA Robot (Dynex Technologies, Worthing, UK). Thresholds for positivity were based on the 97.5th centile of 1500 controls 10: GAD ≥11U/ml, IA2 ≥7.5U/ml and ZnT8 ≥65U/ml.

### Type 1 diabetes genetic risk score

We genotyped by targeted next‐generation sequencing the top nine single nucleotide polymorphisms (SNPs) with the largest effect size on Type 1 diabetes, as previously described 15, including both HLA and non‐HLA regions. The Type 1 diabetes genetic risk score was calculated per individual according to the sum of the number of risk‐increasing alleles across SNPs. Each allele was weighted by its effect on the risk of Type 1 diabetes [ln(odds ratio)], assuming that each risk allele had a log‐additive effect on Type 1 diabetes risk (Table S2).

### Type 1 diabetes cases and controls of European descent

We used Type 1 diabetes case and control individuals from the Wellcome Trust Case Control Consortium (WTCCC) as previously described 15, 23. This cohort includes 1963 individuals with diabetes diagnosed before the age of 17 years and treated with insulin from diagnosis.

### Homozygosity mapping

We defined children born to consanguineous parents as those whose parents were known to be first or second cousins (*n*=37), or where homozygosity mapping calculated directly from next‐generation sequencing off‐target reads using SavvyHomozygosity 24, 25 showed >3% of their genome covered by homozygous regions >3Mb 26. This threshold roughly reflects second cousins in levels of relatedness.

### Statistical analysis

We used chi‐squared analysis to compare proportions (e.g. number of antibody positives) and Wilcoxon's rank‐sum test to compare continuous characteristics (e.g. age of diagnosis) between children with and without monogenic cause. A *P* value < 0.05 was taken to indicate statistical significance. Continuous data are expressed as median and IQR since they were not normally distributed. Logistic regression and receiver‐operating characteristic (ROC) curve analysis were used to measure the discriminatory power of the Type 1 diabetes genetic risk score. Statistical analyses were performed in stata 14 (StataCorp, College Station, TX, USA).

### Ethics approval

The study was approved by the institutional review board of Tehran University of Medical Sciences and Mashhad University of Medical Sciences. All procedures performed in this study were in accordance with the ethical standards of the 1964 Helsinki declaration and its later amendments or comparable ethical standards.

## Results

### Targeted next‐generation sequencing to identify monogenic diabetes

Targeted next‐generation sequencing enabled a genetic diagnosis of monogenic diabetes in six out of 127 children (4.7%; Table 2). Five children had homozygous mutations in genes causing recessive syndromic forms of monogenic diabetes. A homozygous mutation in *WFS1* was identified in three children; mutations in this gene cause Wolfram syndrome, a recessive disorder characterized by childhood‐onset diabetes mellitus, optic atrophy and deafness. One child had a novel frameshift homozygous mutation (c.547del; p.Met183fs) and had other features of Wolfram syndrome, including partial hearing loss in the right ear. The other two children had isolated diabetes and known pathogenic missense homozygous mutations (c.1010C>T; p.Thr337Ile and c.2105G>A; p.Gly702Asp); they had not developed any other features of Wolfram syndrome by age 4 and 9 years, respectively.

**Table 2:** Characteristics of the six children identified with monogenic diabetes

Gene	Mutation; protein effect	Variant type	Gender	Age at diabetes diagnosis, years	Consanguineous	Birth weight, g	Initial treatment	Current treatment	GRS	Type 1 diabetes centile	Other features		Diabetic parent	Diabetic siblings	other family member
***WFS1***	**c.547del; p.(Met183fs)**	**Frameshift**	**Girl**	**1.99**	**No**	**2250**	**Insulin**	**Insulin**	7.002	1	**Partial hearing loss in right ear**		**No**	**No**	**Yes, father's cousin has diabetes, blindness and renal failure**
*WFS1*	c.1010C>T; p.(Thr337Ile)	Missense	Girl	4	First cousin	2700	**Insulin**	**Insulin**	9.101	13.8	Muscle weakness		No	No	No
*WFS1*	c.2105G>A; p.(Gly702Asp)	Missense	Boy	2.47	First cousin	3850	**Insulin**	**Insulin**	8.824	11.3	Nocturia		No	No	No
***SLC19A2***	c.242dup; p.Tyr81*	**Nonsense**	**Boy**	**5**	**First cousin**	**2500**	**Oral**	**Oral**	9.135	14.6	**Developmental delay, anaemia, cardiac defects, low weight, hearing loss, low sight in left eye**		**No**	**No**	**Father's mother, at 54 years old, insulin**
***SLC29A3***	**c.122del; p.(Pro41fs)**	**Frameshift**	**Girl**	**4.42**	**Third cousin**	**3800**	**Insulin**	**Insulin**	8.004	4.1	**No**		**No**	**No**	**Father's mother, at 53 years old, oral treatment. Father's father, at 55 years old, oral treatment**
***GCK***	**c.364‐8T>G**	**Substitution, aberrant effect on splicing**	**Girl**	**0.75**	**No**	**2800**	**No**	**No**	8.151	5.7	**Last HbA**_**1c**_ **50.8 mmol/mol (6.8%)**		**No**	**No**	**Mother's father, diet. Mother's sister and 2 maternal cousins**

GRS, genetic risk score. Novel mutations are in bold.

One child had developmental delay, anaemia, cardiac defects, low weight, hearing loss and low sight in the left eye. These clinical features are consistent with a diagnosis of thiamine‐response megaloblastic anaemia (TRMA), a recessive syndrome caused by mutations in *SLC19A2* 27. The diagnosis of TRMA was confirmed by identifying a homozygous novel *SLC19A2* nonsense mutation (c.242dup; p.Tyr81*).

A pathogenic homozygous frameshift mutation in the *SLC29A3* (c.122del; p.Pro41fs) was identified in another child with isolated diabetes. Mutations in this gene cause H syndrome, characterized by cutaneous findings and multisystem involvement 28, but the child in the present study had no other clinical features of this syndrome at the age of 5.5 years.

The child with a novel substitution heterozygous mutation in *GCK* (c.364‐8T>G) had a phenotype consistent with glucokinase MODY [persistent fasting hyperglycaemia in the range 5.7–6.4 mmol/l, HbA_1c_ 50.8 mmol/mol (6.8%) without treatment and a small postprandial increase in blood glucose evidenced by a 2‐h oral glucose tolerance test value of 7.1 mmol/l]. *In silico* splicing predictions provided evidence to support an aberrant effect on splicing. The variant was also present in the mother and maternal aunt, who were similarly affected. The same *GCK* splicing variant was identified in another Iranian family with clinical features of GCK MODY referred for diagnostic MODY testing to Exeter Molecular Genetics Laboratory. Further information from the family confirmed they came from the same region in North East of Iran.

### Use of Type 1 diabetes genetic risk score to discriminate children with monogenic diabetes from Type 1 diabetes

**Figure 1:**
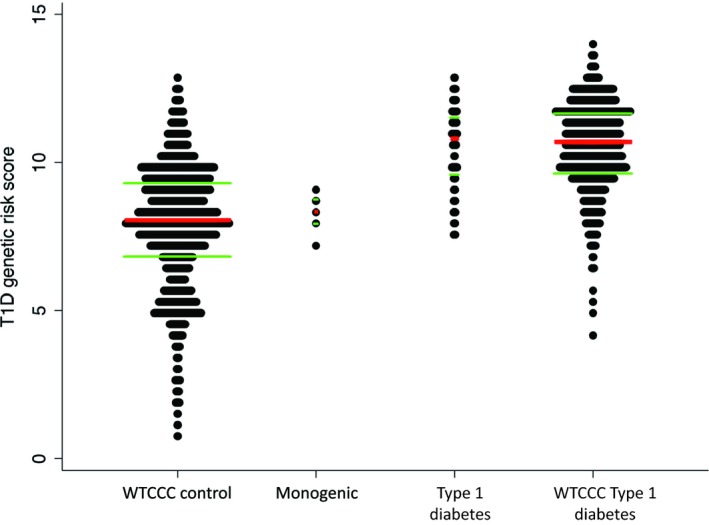
Dot plots of Type 1 diabetes genetic risk score stratified by disease in cases and controls from the Wellcome Trust Case Control Consortium (WTCCC). Type 1 diabetes genetic risk score is higher in children with Type 1 diabetes than in those with confirmed monogenic diabetes. The red central line represents the median and the green upper and lower lines represent the interquartile range.

No pathogenic variants were identified in any of the known monogenic diabetes genes in 121/127 children. Having excluded all known monogenic causes, the most likely diabetes aetiology in this age group is Type 1 diabetes, and this diagnosis was assigned to the 121 children without a monogenic diagnosis. We then assessed the utility of the Type 1 diabetes genetic risk score in indicating the aetiology of diabetes. Children with monogenic diabetes (*n*=6) had a significantly lower median (IQR) Type 1 diabetes genetic risk score than the rest of the cohort [8.4 (8–8.8) vs 10.8 (9.7–11.6)], equivalent to seventh vs 53rd centile in the WTCCC Type 1 diabetes cohort; *P* = 0.005 (Fig. 1)]. Children with mutations (*n*=6) had a similar median (IQR) Type 1 diabetes genetic risk score to that of the WTCCC control cohort [8.4 (8–8.8) vs 8.1 (6.9–9.4)], while the rest of the cohort (*n*=121) had a median (IQR) genetic risk score similar to that of the WTCCC Type 1 diabetes cohort [10.8 (9.7–11.6) vs 10.7 (9.7–11.7); Fig. 1].

**Figure 2:**
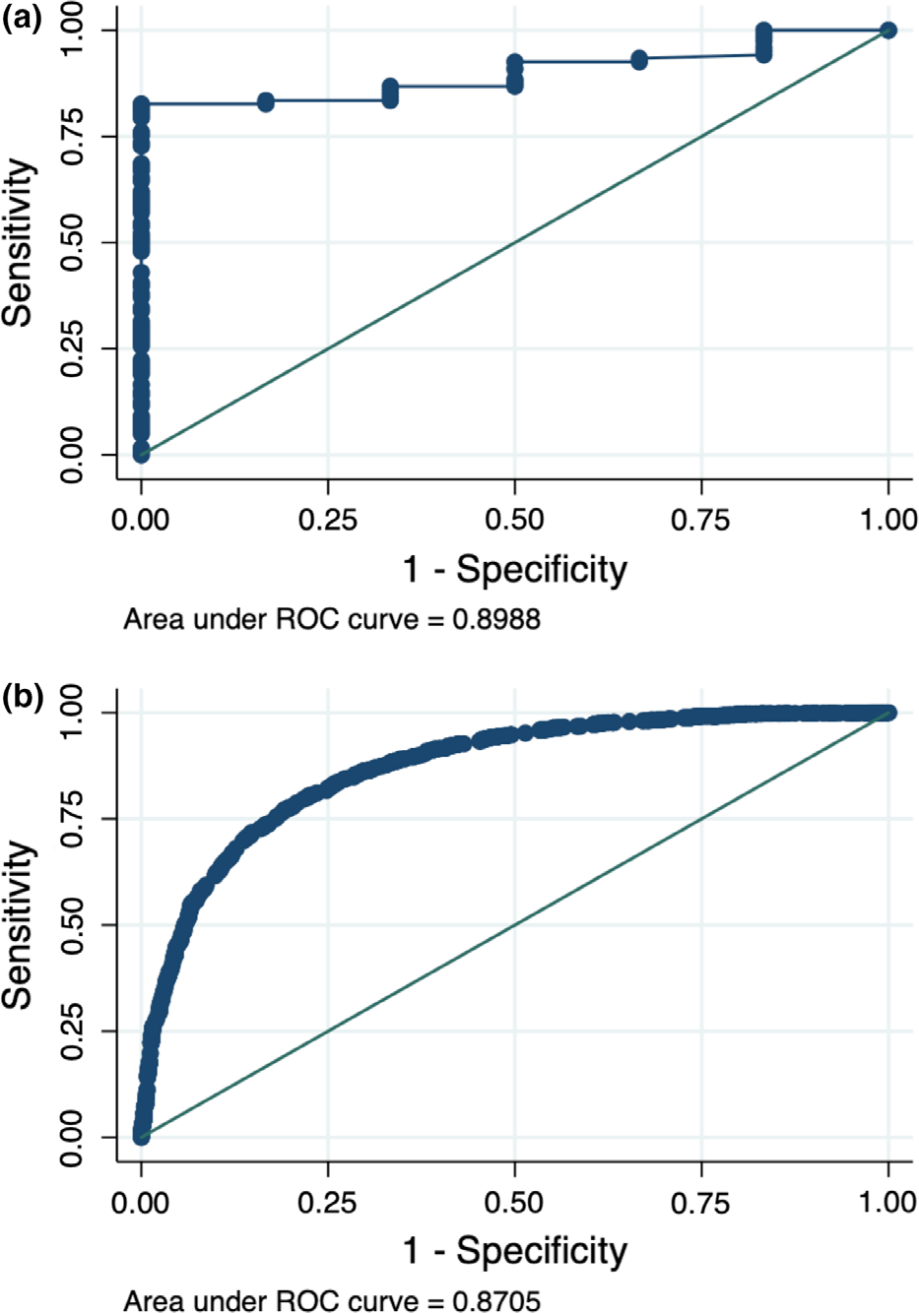
The ability of a nine‐single nucleotide polymorphism (SNP) Type 1 diabetes genetic risk score to discriminate between Type 1 and other types of diabetes in our cohort (a) and in the Wellcome Trust Case Control Consortium study (b). ROC, receiver‐operating characteristic.

The ROC curve analysis showed that the Type 1 diabetes genetic risk score was highly discriminatory between monogenic and Type 1 diabetes in our cohort [area under the ROC curve 0.90 (95% CI 0.83–0.97)], which was similar to the ability of same genetic risk score in the WTCCC Type 1 diabetes cohort to discriminate Type 1 diabetes from controls [area under the ROC curve 0.87 (95% CI 0.86–0.88); Fig. 2]. A Type 1 diabetes genetic risk score threshold of <9.2 (equivalent to 15^th^ centile for Type 1 diabetes) identified all cases of monogenic diabetes (~100% sensitivity and 82% specificity). Using this threshold, we calculated that five children would need to undergo genetic testing to find one case of monogenic diabetes.

### Measuring all three islet antibodies in the diagnosis of Type 1 diabetes

**Figure 3:**
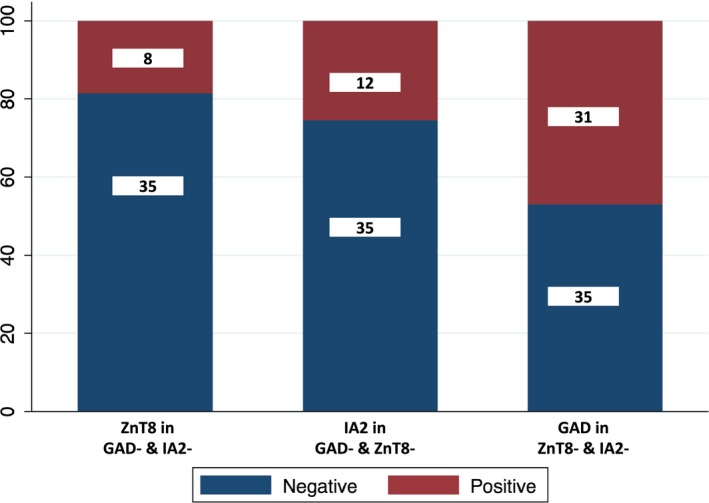
Graph illustrating that measurement of all three islet antibodies can improve the diagnosis of Type 1 diabetes. GAD, glutamic acid decarboxylase; IA2, islet antigen 2; ZnT8, zinc transporter 8.

The analysis of islet autoantibodies was successful for 125/127 children. All children with monogenic diabetes were islet autoantibody‐negative. In 121 children with no mutation, 84 (71%) were positive for at least one antibody and 37 (29%) were negative for all three autoantibodies. A total of 59 were positive for GAD, 39 were positive for IA2 and 31 were positive for ZnT8. Twenty‐one children were positive for any two antibodies and 12 were positive for all three antibodies (Table 1). Children positive for only one antibody included 31 for GAD only, 12 for IA2 only, and eight for ZnT8 only (Fig. 3). Measuring ZnT8 increased the number of auto‐antibody‐positive individuals from 76 to 84, indicating the importance of testing for all three autoantibodies.

### Clinical features in children with monogenic diabetes and those with Type 1 diabetes

Age of diagnosis (*P* = 0.84), consanguinity (*P* = 0.11), last HbA_1c_ value (*P* = 0.76), syndromic features (*P* = 0.36), gender (*P* = 0.70), duration of diabetes (*P* = 0.51) and proportion of children with a parent who had diabetes (*P* = 0.51) were similar in children with monogenic diabetes and the remainder of the cohort (Table 1). Children with Type 1 diabetes were significantly more likely to be insulin‐treated at the time of diabetes diagnosis and at the time of recruitment into the study.

## Discussion

We have provided the first evidence to suggest that the Type 1 diabetes genetic risk score may help to distinguish monogenic diabetes from Type 1 diabetes in an Iranian population with a large number of consanguineous unions. Six children with monogenic diabetes had a lower Type 1 diabetes genetic risk score than those with probable Type 1 diabetes. The age at diagnosis, consanguinity, presence of other symptoms and parental diabetes status were similar in the two groups and did not aid discrimination, highlighting the need for a non‐clinical marker for selecting children for monogenic diabetes testing.

The present study provides evidence that the Type 1 diabetes genetic risk score could be used in clinical practice in non‐European ethnic groups, such as Iranians. Iran is a Middle Eastern country with a high prevalence of diabetes (11.1%) 29, obesity 30 and consanguinity (37.4% of marriages 31). The features of diabetes in Iran means people with diabetes are likely to be misclassified because those with Type 1 diabetes may be overweight, and family history may not distinguish monogenic forms. High rates of consanguinity mean that many undiscovered monogenic recessive forms of diabetes may exist in the population. All these factors suggest standard clinical criteria used in Europe may not translate well to a Middle Eastern setting.

We propose that GAD, IA2 and ZnT8 autoantibody testing, in combination with the Type 1 diabetes genetic risk score, could be used to prioritize individuals for genetic testing. There were 13 children in our cohort who were islet autoantibody‐negative and had a Type 1 diabetes genetic risk score below that of the 15^th^ centile of European people with Type 1 diabetes. Among these children, we confirmed monogenic diabetes in six; therefore, in three islet antibody‐negative children with a genetic risk score <15th centile, we need to test two cases to obtain one monogenic diabetes diagnosis (~50% identification rate). Studies of larger numbers of children in this age range would enable us to use islet autoantibodies and Type 1 diabetes genetic risk score to provide a screening pathway for monogenic diabetes in this population.

Although the number of individuals with monogenic diabetes in the present cohort was low, to our knowledge, this is the first report of using targeted next‐generation sequencing to diagnose monogenic diabetes in young Iranian children described in the literature to date. A diagnosis of monogenic diabetes was confirmed in six children, with five (83%) having a recessive, syndromic subtype. Mutations in *WFS1* (associated with Wolfram syndrome) accounted for 50% of the monogenic diabetes cases. This is significantly higher than the 0.1% estimate of Wolfram syndrome prevalence in a European paediatric population 32. Wolfram syndrome is recessively inherited and the median age of diabetes diagnosis in that population was 6 years (range 3 weeks to 17 years) 33. The high prevalence was therefore not unexpected given the consanguineous nature of the present cohort and the age range of diabetes diagnosis.

It is likely that in a similar age group in a non‐consanguineous European population, the overall diagnostic yield from monogenic diabetes testing would be lower owing to the absence of rare recessive subtypes. In the UK population, *HNF1A* MODY is the most common cause of monogenic diabetes outside of the neonatal period 34. Because of the progressive nature of the β‐cell defect, those with *HNF1A* MODY are normoglycaemic at birth and early childhood but develop diabetes as teenagers and early adults 35. Fewer than 1% of *HNF1A* MODY cases are diagnosed under the age of 10 years 36. *GCK* MODY is the second most common subtype; people who have this subtype are typically asymptomatic and are often diagnosed incidentally when fasting blood glucose testing is undertaken for other purposes (e.g. during pregnancy, illness or routine medical screening) 37; therefore, almost all monogenic diabetes in the UK diagnosed between the ages of 1 to 5 years would be *GCK* MODY and only very rarely attributable to recessive syndromic subtypes.

The Type 1 diabetes genetic risk score could enable the diagnosis of syndromic forms of monogenic diabetes when clinical features are not present. Among three children with a mutation in *WFS1*, the extra‐pancreatic features associated with Wolfram syndrome were present in only one child. This is probably attributable to the early genetic diagnosis when only diabetes is present and before the development of other associated features, such as optic atrophy and deafness. The child with a mutation in *SLC29A3* had no clinical manifestations attributed to H syndrome. The mutations in *SLC29A3* have also been detected in children with mild manifestations and our findings in this child indicate that the prevalence of H syndrome is likely to be underestimated as a result of undiagnosed mild cases 28. The child with a mutation in *SLC19A2* had developed other features of thiamine‐responsive megaloblastic anaemia syndrome, also known as Roger's syndrome, including anaemia, cardiac defects and deafness; however, studies of other cases suggest diabetes can be isolated and present before the appearance of other features 38, 39. Prompt diagnosis is essential as more than half of the individuals with follow‐up data benefitted from early treatment with thiamine, with some individuals becoming insulin‐independent 39.

We showed for the first time that ZnT8 antibodies could be detected in 18.6% of Iranian children with a Type 1 diabetes phenotype who lack GAD and IA2 antibodies. This is very similar to observations in European children with Type 1 diabetes, where testing for ZnT8 antibodies increased the number of individuals positive for only one autoantibody by 14–18% 12, 40. This finding suggests that measuring ZnT8 antibodies in addition to GAD and IA2 antibodies could increase the sensitivity and specificity to detect the presence of an immune‐mediated disease process.

The present study has some limitations. First, we were unable to assay serum C‐peptide in our children to confirm diagnosis of Type 1 diabetes; however, the present cohort included children aged 9 months to 5 years and C‐peptide measurement is only discriminative 3 to 5 years after diagnosis because of the ‘honeymoon period’ 41.

Second, antibody testing was performed at time of genetic testing and not at the time of diagnosis. Previous studies have shown that GAD, IA2 and ZnT8 antibody titres do not fall significantly in the first 2 years after diagnosis 42, 43 and our cohort had a median diabetes duration of 1.6 years; however, we acknowledge that antibodies may have been present in the children with longer diabetes duration but may have been no longer in circulation.

Third, for technical reasons of genotyping, we only used nine common SNPs with the highest risk alleles for maximum discrimination between Type 1 diabetes and other subtypes. However, it has been shown that other SNPs do not substantially improve discriminatory ability as a result of being rare or having a subtle effect size 15.

Fourth, our cohort included a small number of children with monogenic diabetes (*n* = 6) and there will be a degree of uncertainty in the estimates of sensitivity and specificity of the genetic risk score to discriminate monogenic diabetes from Type 1 diabetes. The utility of antibodies and genetic risk score was determined in a cohort of children with age of diagnosis of diabetes between 9 months and 5 years. In this age group, Type 1 diabetes or rare recessive monogenic forms will be the only subtypes, and Type 1 diabetes genetic risk score and antibodies are always likely to be discriminative. This age range would exclude more common subtypes of monogenic diabetes, as discussed above, and therefore does not inform about MODY vs Type 1 diabetes.

Further work is needed to validate the robustness of the discriminative ability of the Type 1 diabetes genetic risk score and antibodies in a larger cohort of people with diabetes diagnosed up to the age of 30 or 35 years. This would enable the genetic diagnosis of more common, dominant forms of MODY and provide the power to test the ability of antibodies and genetic risk score to distinguish Type 1 from both MODY and Type 2 diabetes in non‐European populations.

The final limitation is that we used a genetic risk score that was developed in British European individuals. It is possible that population stratification, ethnicity and higher rates of consanguinity may result in differences in the underlying risk allele frequencies between European and Iranian populations. No large‐scale genome‐wide SNP genotyping or sequencing has been performed in the Iranian population and the true frequency of the Type 1 diabetes risk alleles used in the genetic risk score are not known. We also used odds ratios derived from Europeans for the Type 1 diabetes genetic risk score. The use of large genome‐wide association studies to generate the weights in the Type 1 diabetes genetic risk score means the odds ratios are precise for a European population. It is reassuring that the genetic risk score of Iranian people with Type 1 diabetes did not differ significantly from Europeans. European population‐derived Type 1 diabetes risk alleles have also been shown to discriminate Type 1 diabetes in Hispanic and African populations 44, 45. Further work is needed to try and define genetic relationships in a large Iranian cohort with Type 1 diabetes to generate an Iranian‐specific genetic risk score; however, a critical issue is the power required to do this and, without large sample sizes, it is possible that a genetic risk score defined in a small cohort (e.g. <1000 cases) may not improve discrimination of Type 1 diabetes.

In conclusion, we have demonstrated that the Type 1 diabetes genetic risk score has the potential to discriminate Type 1 from monogenic diabetes in children diagnosed between the ages of 9 months and 5 years from an Iranian population with a large number of consanguineous unions. Genetic risk score in combination with GAD, IA2 and ZnT8 autoantibody testing could be used to identify people with a higher probability of having monogenic diabetes who could then undergo genetic testing. Identification of these individuals could potentially reduce the cost of treatment and improve the management of their clinical course.

## Funding sources

This work was supported by the Wellcome Trust [108101/Z/15/Z]. H.Y. is funded by a Diabetes UK RD Lawrence fellowship (grant: 17/0005594). A.T.H. and S.E. are Wellcome Trust Senior Investigators and A.T.H. is an National Institute for Health Research senior investigator.

## Competing interests

None declared.

## Ethical Approval

The study was approved by the institutional review board of Tehran University of Medical Sciences and Mashhad University of Medical Sciences, and informed consent was obtained from all participants. This research study was conducted in accordance with the guidelines of the Declaration of Helsinki.

## Supporting information


**Table S1** Genes sequenced by the targeted next‐generation sequencing assay.
**Table S2** Type 1 diabetes SNPs included in the genetic risk score with weights. Effect allele is the risk increasing allele on the positive strand.
