# Supplementary material for: Type 1 diabetes genetic risk score discriminates between monogenic and Type 1 diabetes in children diagnosed at the age of <5 years in the Iranian population
Source: Diabet Med. 2019 Jul 25;36(12):1694–702. doi: 10.1111/dme.14071 (PMC7027759; doi:10.1111/dme.14071)
Supplement: Supplementary file 1 — Table S1 Genes sequenced by the targeted next‐generation sequencing assay. Table S2 Type 1 diabetes SNPs included in the genetic risk score with weights. Effect allele is the risk increasing allele on the positive strand. [file DME-36-1694-s001.docx]

**Supplementary Table 1**: Genes sequenced by the targeted NGS assay.

| Gene (OMIM) | Genbank Reference Sequence | Phenotype | OMIM | Inheritance | References |
| --- | --- | --- | --- | --- | --- |
| *ABCC8*  [600509](http://omim.org/entry/600509) | NM_001287174 | Permanent neonatal diabetes | [606176](http://omim.org/entry/606176) | Dominant  (often *de novo*) or recessive | [Proks *et al* 2006 Hum Mol Genet 15: 1793-1800](http://www.ncbi.nlm.nih.gov/pubmed/?term=Proks+HMG+2006)  [Babenko *et al* 2006 N Engl J Med 355: 456-466](http://www.ncbi.nlm.nih.gov/pubmed/?term=Babenko+2006+NEJM)  [Ellard *et al* 2007 Am J Hum Genet 81: 375-382](http://www.ncbi.nlm.nih.gov/pubmed/?term=Ellard+et+al+2007+Am+J+Hum+Genet+81%2C+375-382) |
|  |  | Transient neonatal diabetes | [610374](http://omim.org/entry/610374) | Dominant  (often *de novo*) or recessive | [Babenko *et al* 2006 N Engl J Med 355: 456-466](http://www.ncbi.nlm.nih.gov/pubmed/?term=Babenko+2006+NEJM) |
|  |  | MODY | [610374](http://omim.org/entry/610374) | Dominant | [Bowman *et al* 2012 Diabetologia 55: 123-127](http://www.ncbi.nlm.nih.gov/pubmed/?term=Bowman+Diabetologia+2012)  [Riveline *et al* 2012 Diabetes Care 35: 248-251](http://www.ncbi.nlm.nih.gov/pubmed/?term=Riveline+et+al+2012+Diabetes+Care+35%2C+248-251) |
| *AGPAT2*  [603100](https://www.omim.org/entry/603100) | NM_006412 | Congenital generalised lipodystrophy | [608594](https://www.omim.org/entry/608594) | Recessive | [Agarwal *et al* 2002 Nat Genet 31: 21-23](https://www.ncbi.nlm.nih.gov/pubmed/11967537) |
| *AIRE*  [607358](https://www.omim.org/entry/607358) | NM_000383 | Type 1 autoimmune polyendocrinopathy syndrome and autoimmune diabetes | [240300](https://www.omim.org/entry/240300) | Dominant  (often *de novo*) or recessive | [Finnish-German APECED Consortium 1997 Nat Genet 17: 399-403](https://www.ncbi.nlm.nih.gov/pubmed/9398840) |
| *AKT2*  [164731](https://www.omim.org/entry/164731) | NM_001626 | Lipodystrophy and severe insulin resistance | Not assigned | Dominant | [George *et al* 2004 Science 304: 1325-1328](https://www.ncbi.nlm.nih.gov/pubmed/15166380)  [Semple *et al* 2009 J Clin Invest 119: 315-322](https://www.ncbi.nlm.nih.gov/pubmed/19164855) |
| *APPL1*  [604299](https://www.omim.org/entry/604299) | NM_012096 | MODY | [616511](https://www.omim.org/entry/616511) | Dominant | [Prudente *et al* 2015 Am J Hum Genet 97: 177-185](https://www.ncbi.nlm.nih.gov/pubmed/26073777) |
| *BSCL2*  [606158](http://www.omim.org/entry/606158) | NM_032667 | Congenital generalised lipodystrophy, severe insulin resistance and diabetes | [269700](http://www.omim.org/entry/269700) | Recessive | [Magre *et al* 2001 Nat Genet 28: 365-370](http://www.ncbi.nlm.nih.gov/pubmed/11479539) |
| *CTLA4*  [123890](https://www.omim.org/entry/123890) | NM_005214 | Type V autoimmune lymphoproliferative syndrome and autoimmune diabetes | [616100](https://www.omim.org/entry/616100) | Dominant | [Schubert *et al* 2014 Nature Med 20: 1410-1416](https://www.ncbi.nlm.nih.gov/pubmed/25329329)  [Kuehn *et al* 2014 Science 345: 1623-1627](https://www.ncbi.nlm.nih.gov/pubmed/25213377) |
| *CEL*  [114840](http://omim.org/entry/114840) | NM_001807 | MODY | [609812](http://omim.org/entry/609812) | Dominant | [Raeder *et al* 2006 Nat Genet 38: 54-62](http://www.ncbi.nlm.nih.gov/pubmed/?term=Raeder+et+al+2006+Nat+Genet+38%2C+54-62)  [Torsvik *et al* 2010 Hum Genet 127: 55-64](http://www.ncbi.nlm.nih.gov/pubmed/?term=Torsvik+Hum+Genet+2010)  [Raeder *et al* 2013 PLoS One 8: e60229](http://www.ncbi.nlm.nih.gov/pubmed/23565203) |
| *CISD2*  [611507](http://www.omim.org/entry/611507) | NM_001008388 | Wolfram Syndrome 2 (diabetes mellitus, hearing loss, optic atrophy and defective platelet aggregation). | [604928](http://www.omim.org/entry/604928) | Recessive | [Amr *et al* 2007 Amr J Hum Genet 81: 673-683](http://www.ncbi.nlm.nih.gov/pubmed/17846994) |
| *COQ2*  [609825](https://www.omim.org/entry/609825) | NM_015697 | Coenzyme Q10 deficiency, primary, 1 (hyperglycaemia reported) | [607426](https://www.omim.org/entry/607426) | Recessive | [Quinzii *et al* 2006 Am J Hum Genet 78: 345-349](https://www.ncbi.nlm.nih.gov/pubmed/16400613) |
| *COQ9*  [612837](https://www.omim.org/entry/612837) | NM_020312 | Coenzyme Q10 deficiency, primary, 5 (hyperglycaemia reported) | [614654](https://www.omim.org/entry/614654) | Recessive | [Duncan *et a*l 2009 Am J Hum Genet 84: 558-566](https://www.ncbi.nlm.nih.gov/pubmed/19375058) |
| *DCAF17*  [612515](https://www.omim.org/entry/612515) | NM_025000 | Woodhouse-Sakati syndrome | [241080](https://www.omim.org/entry/241080) | Recessive | [Alazami *et al* 2008 Am J Hum Genet 83: 684-691](https://www.omim.org/entry/612515) |
| *DNAJC3*  [601184](https://www.omim.org/entry/601184) | NM_006260 | Autosomal recessive juvenile-onset diabetes with central and peripheral neurodegeneration | [616192](https://www.omim.org/entry/616192) | Recessive | [Synofzik *et al* 2014 Am J Hum Genet 95: 689-697](https://www.ncbi.nlm.nih.gov/pubmed/25466870) |
| *DYRK1B*  [604556](https://www.omim.org/entry/604556) | NM_004714 | Diabetes and metabolic syndrome | [615812](https://www.omim.org/entry/615812) | Dominant | [Keramati *et al* 2014 New Eng J Med 370: 1909-1919](https://www.ncbi.nlm.nih.gov/pubmed/24827035) |
| *EIF2AK3*  [604032](http://omim.org/entry/604032) | NM_004836 | Wolcott-Rallison syndrome | [226980](http://omim.org/entry/226980) | Recessive | [Delephine *et al* 2000 Nat Genet 25: 406-409](http://www.ncbi.nlm.nih.gov/pubmed/?term=Delephine+Nature+Genetics+2000) |
| *EIF2S3*  [300161](https://www.omim.org/entry/300161) | NM_001415 | Borck type of X-linked syndromic mental retardation and neonatal diabetes | [300987](https://www.omim.org/entry/300987) | X-Linked Recessive | [Moortgat *et al* 2016 Am J Med Genet 170A: 2927-2933](https://www.ncbi.nlm.nih.gov/pubmed/27333055) |
| *FOXP3*  [300292](http://omim.org/entry/300292) | NM_014009 | Immunodysregulation, polyendocrinopathy, and enteropathy, X-linked syndrome (IPEX) | [304790](http://omim.org/entry/304790) | X-Linked Recessive | [Wildin *et al* 2001 Nat Genet 1: 18-20](http://www.ncbi.nlm.nih.gov/pubmed/?term=Wildin+et+al+2001+Nat+Genet+1%2C+18-20)  [Bennett *et al* 2001 Nat Genet 27: 20-21](http://www.ncbi.nlm.nih.gov/pubmed/11137993) |
| *GATA4*  [600576](http://omim.org/entry/600576) | NM_002052 | Permanent neonatal diabetes with pancreatic agenesis and congenital heart defects | Not assigned | Dominant  (often *de novo*) | [D’Amato *et al* 2010 Diabet Med 27: 1195-1200](http://www.ncbi.nlm.nih.gov/pubmed/?term=D%E2%80%99Amato+Diabet+Med+2010) |
| *GATA6*  [601656](http://omim.org/entry/601656) | NM_005257 | Permanent neonatal diabetes with pancreatic agenesis and congenital heart defects | [600001](http://omim.org/entry/600001) | Dominant  (often *de novo*) | [Lango Allen *et al* 2011 Nat Genet 44: 20-22](http://www.ncbi.nlm.nih.gov/pubmed/22158542)  [De Franco *et al* 2013 Diabetes 62: 993-997](http://www.ncbi.nlm.nih.gov/pubmed/23223019) |
| *GCK*  [138079](http://omim.org/entry/138079) | NM_000162 | Permanent neonatal diabetes | [606176](http://omim.org/entry/606176) | Recessive | [Njolstad *et al* 2001 N Engl J Med 344: 1588-1592](http://www.ncbi.nlm.nih.gov/pubmed/11372010)  [Gloyn *et al* 2002 Diabetologia 45: 290](http://www.ncbi.nlm.nih.gov/pubmed/11942315)  [Osbak *et al* 2009 Hum Mutat 30: 1512-1526](http://www.ncbi.nlm.nih.gov/pubmed/?term=osbak+2009+GCK) |
|  |  | MODY | [125851](http://omim.org/entry/125851) | Dominant | [Vionnet *et al* 1992 Nature 356: 721-722](http://www.ncbi.nlm.nih.gov/pubmed/?term=Vionnet+et+al+1992+Nature+356%2C+721-722)  [Velho *et al* 1997 Diabetologia 40: 217-224](http://www.ncbi.nlm.nih.gov/pubmed/?term=velho+diabetologia+1997)  [Osbak *et al* 2009 Hum Mutat 30: 1512-1526](http://www.ncbi.nlm.nih.gov/pubmed/?term=osbak+2009+GCK) |
| *GLIS3*  [610192](http://omim.org/entry/610192) | NM_001042413 | Permanent neonatal diabetes with congenital hypothyroidism | [610199](http://omim.org/entry/610199) | Recessive | [Senee *et al* 2006 Nat Genet 38: 682-687](http://www.ncbi.nlm.nih.gov/pubmed/?term=Senee+2006+Nature+Genetics)  [Dimitri *et al* 2011 Eur J Endocrinol 164: 437-443](http://www.ncbi.nlm.nih.gov/pubmed/?term=Dimitri+EJE+2011) |
| *HNF1A*  [142410](http://omim.org/entry/142410) | NM_000545 | MODY | [600496](http://omim.org/entry/600496) | Dominant | [Yamagata *et al* 1996 Nature 384: 455-458](http://www.ncbi.nlm.nih.gov/pubmed/?term=Yamagata+et+al+1996+Nature+384%2C+455-458)  [Frayling *et al* 1997 Diabetes 46: 720-725](http://www.ncbi.nlm.nih.gov/pubmed/9075818)  [Colclough *et al* 2013 Hum Mutat 34: 669-685](http://www.ncbi.nlm.nih.gov/pubmed/23348805) |
| *HNF1B*  [189907](http://omim.org/entry/189907) | NM_000458 | Renal Cysts and Diabetes syndrome (RCAD) | [137920](http://omim.org/entry/137920) | Dominant  (often *de novo*) | [Horikawa *et al* 1997 Nat Genet 17: 384-385](http://www.ncbi.nlm.nih.gov/pubmed/9398836)  [Yorifuji *et al* 2004 J Clin Endocrinol Metab 89: 2905-2908](http://www.ncbi.nlm.nih.gov/pubmed/?term=Yorifuji+et+al+2004+J+Clin+Endocrinol+Metab+89%2C+2905-2908)  [Edghill *et al* 2006 J Med Genet 43: 84-90](http://www.ncbi.nlm.nih.gov/pubmed/?term=edghill+J+Med+Genet+2006)  [Bellanne-Chantelot *et al* 2005 Diabetes 54: 3126-3132](http://www.ncbi.nlm.nih.gov/pubmed/16249435) |
| *HNF4A*  [600281](http://omim.org/entry/600281) | NM_175914 | MODY | [125850](http://omim.org/entry/125850) | Dominant | [Yamagata *et al* 1996 Nature 384: 458-460](http://www.ncbi.nlm.nih.gov/pubmed/?term=Yamagata+et+al+1996+Nature+384%2C+458-460)  [Bulman *et al* 1997 Diabetologia 40: 859-862](http://www.ncbi.nlm.nih.gov/pubmed/?term=bulman+diabetologia+1997)  [Colclough *et al* 2013 Hum Mutat 34: 669-685](http://www.ncbi.nlm.nih.gov/pubmed/23348805) |
| *IER3IP1*  [609382](http://omim.org/entry/609382) | NM_016097 | microcephaly, epilepsy, and diabetes syndrome (MEDS) | [614231](http://omim.org/entry/614231) | Recessive | [Poulton et al 2011 Am J Hum Genet 89: 265-276](http://www.ncbi.nlm.nih.gov/pubmed/?term=Poulton+et+al+2011+Am+J+Hum+Genet+89%2C+265-276)  [Abdel-Salam *et al* 2012 Am J Med Genet A 158A: 2788-2796](http://www.ncbi.nlm.nih.gov/pubmed/22991235) |
| *IL2RA*  [147730](http://omim.org/entry/147730) | NM_000417 | Immunodeficiency 41 with lymphoproliferation, autoimmunity and autoimmune diabetes | [606367](http://omim.org/entry/606367) | Recessive | [Caudy *et al* 2007 J Allergy Clin Immunol 119: 482-487](https://www.ncbi.nlm.nih.gov/pubmed/17196245) |
| *INS*  [176730](http://omim.org/entry/176730) | NM_001185098 | Permanent neonatal diabetes | [606176](http://omim.org/entry/606176) | Dominant  (often *de novo*) or recessive | [Stoy *et al* 2007 Proc Natl Acad Sci USA 18: 15040-15044](http://www.ncbi.nlm.nih.gov/pubmed/?term=Stoy+PNAS+2007)  [Edghill *et al* 2008 Diabetes 57: 1034-1042](http://www.ncbi.nlm.nih.gov/pubmed/18162506) |
|  |  | Transient neonatal diabetes | Not assigned | Dominant  (often *de novo*) or recessive | [Garin *et al* 2010 Proc Natl Acad Sci 107: 3105-3110](http://www.ncbi.nlm.nih.gov/pubmed/20133622) |
|  |  | MODY | [613370](http://omim.org/entry/613370) | Dominant | [Edghill *et al* 2008 Diabetes 57: 1034-1042](http://www.ncbi.nlm.nih.gov/pubmed/18162506)  [Molven *et al* 2008 Diabetes 57: 1131-1135](http://www.ncbi.nlm.nih.gov/pubmed/?term=Molven+Diabetes+2008+INS) |
| *INSR*  [147670](http://www.omim.org/entry/147670) | NM_000208 | Severe insulin resistance | [610549](http://www.omim.org/entry/610549) | Dominant | [Odawara *et al* 1989 Science 245: 66-68](http://www.ncbi.nlm.nih.gov/pubmed/2544998) |
| *ITCH*  [606409](https://www.omim.org/entry/606409) | NM_001257138 | Multisystem autoimmune disease with facial dysmorphism and autoimmune diabetes | [613385](https://www.omim.org/entry/613385) | Recessive | [Lohr *et al* 2010 Am J Hum Genet 86: 447-453](https://www.ncbi.nlm.nih.gov/pubmed/20170897) |
| *JAK1*  [147795](https://www.omim.org/entry/147795) | NM_002227 | Immune dysregulatory and hypereosinophilic syndrome | Not assigned | Dominant | [Del Bel *et al* 2017 J Allergy Clin Immunol 139: 2016-2020](https://www.ncbi.nlm.nih.gov/pubmed/28111307) |
| *KCNJ11*  [600937](http://omim.org/entry/600937) | NM_000525 | Permanent neonatal diabetes | [606176](http://omim.org/entry/606176) | Dominant  (often *de novo*) | [Gloyn *et al* 2004 N Engl J Med 350: 1838-1849](http://www.ncbi.nlm.nih.gov/pubmed/?term=Gloyn+2004+NEJM) |
|  |  | Transient neonatal diabetes | [610582](http://omim.org/entry/610582?search=610582&highlight=610582) | Dominant  (often *de novo*) | [Yorifuji *et al* 2005 J Clin Endocrinol Metab 90: 3174-3178](http://www.ncbi.nlm.nih.gov/pubmed/?term=Yorifuji+et+al+2005+J+Clin+Endocrinol+Metab+90%2C+3174-3178)  [Gloyn *et al* 2005 Hum Mol Genet 14: 925-934](http://www.ncbi.nlm.nih.gov/pubmed/?term=Gloyn+2005+HMG)  [Suzuki *et al* 2007 J Clin Endocrinol Metab 92: 3979-3985](http://www.ncbi.nlm.nih.gov/pubmed/17635943) |
|  |  | MODY | [616329](http://www.omim.org/entry/616329) | Dominant | [Yorifuji *et al* 2005 J Clin Endocrinol Metab 90: 3174-3178](http://www.ncbi.nlm.nih.gov/pubmed/?term=Yorifuji+et+al+2005+J+Clin+Endocrinol+Metab+90%2C+3174-3178)  [Bonnefond *et al* 2012 PLoS One 7: e37423](http://www.ncbi.nlm.nih.gov/pubmed/22701567) |
| *LMNA*  [150330](http://omim.org/entry/150330) | NM_170707 | Familial Partial Lipodystrophy (FPLD2) and insulin resistance | [151660](http://omim.org/entry/151660) | Dominant | [Cao *et al* 2000 Hum Mol Genet 1: 109-112](http://www.ncbi.nlm.nih.gov/pubmed/?term=Cao+et+al+2000+Hum+Mol+Genet+1%2C+109-112)  [Shackleton *et al* 2000 Nat Genet 24: 153-156](http://www.ncbi.nlm.nih.gov/pubmed/?term=Shackleton+Nat+Genet+2000)  [Speckman *et al* 2000 Am J Hum Genet 66: 1192-1198](http://www.ncbi.nlm.nih.gov/pubmed/?term=Bowman+Diabetologia+2012) |
| *LPL*  [609708](https://www.omim.org/entry/609708) | NM_00237 | Lipoprotein lipase deficiency and transient neonatal diabetes | [238600](https://www.omim.org/entry/238600) | Recessive | [Raupp *et al* 2002 J Inherit Metab Dis 25: 413-414](https://www.ncbi.nlm.nih.gov/pubmed/12408192) |
| *LRBA*  [606453](http://omim.org/entry/606453) | NM_001199282 | Immunodysregulation and autoimmune diabetes | [606453](http://omim.org/entry/614700) | Recessive | [Charbonnier *et al* 2015 J Allergy Clin Immunol 135: 217-227](http://www.ncbi.nlm.nih.gov/pubmed/25468195)  [Schreiner *et al* 2016 J Clin Endocrinol Metab 101: 898-904](http://www.ncbi.nlm.nih.gov/pubmed/26745254) |
| *MNX1*  [142994](http://www.omim.org/entry/142994) | NM_005515 | Neonatal diabetes & IUGR | Not assigned | Recessive | [Flanagan *et al* 2014 Cell Metab 19: 146-154](http://www.ncbi.nlm.nih.gov/pubmed/24411943) |
| *MTTL1* g.3243A>G  [590050](http://omim.org/entry/590050) | NC_012920 | Maternally inherited diabetes and deafness (MIDD) | [520000](http://omim.org/entry/520000?search=diabetes%20deafness&highlight=deafness%20diabete%20deaf%20diabetic) | Mitochondrial | [Van den Ouweland *et al* 1992 Nat Genet 1: 368-371](http://www.ncbi.nlm.nih.gov/pubmed/?term=Van+den+Ouweland+et+al+1992+Nat+Genet+1%2C+368-371)  [Murphy *et al* 2008 Diabet Med 25: 383-399](http://www.ncbi.nlm.nih.gov/pubmed/?term=murphy+MIDD) |
| *NEUROD1*  [601724](http://omim.org/entry/601724) | NM_002500 | Permanent neonatal diabetes and neurological abnormalities | Not assigned | Recessive | [Rubio-Cabezas *et al* 2010 Diabetes 162: 987-992](http://www.ncbi.nlm.nih.gov/pubmed/20573748) |
|  |  | MODY | [606394](http://omim.org/entry/606394) | Dominant | [Malecki *et al* 1999 Nat Genet 23: 323-328](http://www.ncbi.nlm.nih.gov/pubmed/?term=Malecki+Nat+Genet+1999) |
| *NEUROG3*  [604882](http://omim.org/entry/604882) | NM_020999 | Permanent neonatal diabetes with congenital malabsorptive diarrhoea | [610370](http://omim.org/entry/610370) | Recessive | [Rubio-Cabezas *et al* 2011 Diabetes 60: 1349-1353](http://www.ncbi.nlm.nih.gov/pubmed/21378176) |
| *NKX2-2*  [604612](http://www.omim.org/entry/604612) | NM_002509 | Neonatal diabetes and developmental delay | Not assigned | Recessive | [Flanagan *et al* 2014 Cell Metab 19: 146-154](http://www.ncbi.nlm.nih.gov/pubmed/24411943) |
| *PAX6*  [607108](http://www.omim.org/entry/607108) | NM_001604 | Aniridia and impaired glucose tolerance | [106210](http://www.omim.org/entry/106210) | Dominant | [Yasuda *et al* 2002 Diabetes 51: 224-230](http://www.ncbi.nlm.nih.gov/pubmed/11756345)  [Nishi](http://www.ncbi.nlm.nih.gov/pubmed/15842522) *[et al](http://www.ncbi.nlm.nih.gov/pubmed/15842522)* [2005 Diabet Med 22: 641-644](http://www.ncbi.nlm.nih.gov/pubmed/15842522)  [Osawa *et al* 2015 J Diabetes Investig 6: 105-106](http://www.ncbi.nlm.nih.gov/pubmed/25621140) |
| *PCBD1*  [126090](http://omim.org/entry/126090) | NM_000281 | Hyperphenylalaninemia and diabetes | [264070](https://omim.org/entry/264070) | Recessive | [Simaite *et al* 2014 Diabetes 63: 3557-3564](http://www.ncbi.nlm.nih.gov/pubmed/24848070)  [Ferre *et al* 2014 J Am Soc Nephrol 25: 574-586](http://www.ncbi.nlm.nih.gov/pubmed/24204001) |
| *PDX1*  [600733](http://omim.org/entry/600733) | NM_000209 | Permanent neonatal diabetes +/- pancreatic agenesis | [260370](http://omim.org/entry/260370) | Recessive | [Stoffers *et al* 1997 Nat Genet 15: 106-110](http://www.ncbi.nlm.nih.gov/pubmed/?term=Stoffers+et+al+1997+Nat+Genet+15%2C+106-110)  [Thomas *et al* 2009 Pediatr Diabetes 10: 492-496](http://www.ncbi.nlm.nih.gov/pubmed/19496967)  [De Franco *et al* 2013 Diabet Med 30: e197-200](http://www.ncbi.nlm.nih.gov/pubmed/?term=De+Franco+Diabet+Med+2013) |
|  |  | MODY | [606392](http://omim.org/entry/606392) | Dominant | [Stoffers *et al* 1997 Nat Genet 17: 138-139](http://www.ncbi.nlm.nih.gov/pubmed/9326926) |
| *PIK3R1*  [171833](https://www.omim.org/entry/171833) | NM_181523 | SHORT syndrome | [269880](https://www.omim.org/entry/269880) | Dominant | [Dyment *et al* 2013 Am J Hum Genet 93: 158-166](https://www.ncbi.nlm.nih.gov/pubmed/23810382) |
| *PLIN1*  [170290](http://omim.org/entry/170290) | NM_002666 | Familial Partial Lipodystrophy (FPLD4) and insulin resistance | [613877](http://omim.org/entry/613877) | Dominant | [Gandotra *et al* 2011 N Engl J Med 364: 740-748](http://www.ncbi.nlm.nih.gov/pubmed/21345103) |
| *POLD1*  [174761](http://omim.org/entry/174761) | NM_002691 | Mandibular hypoplasia, deafness, progeroid features, and lipodystrophy (MDPL) syndrome | [615381](http://omim.org/entry/615381) | Dominant  (*de novo*) | [Weedon *et al* 2013 Mat Genet 45: 947-950](http://www.ncbi.nlm.nih.gov/pubmed/23770608) |
| *PPARG*  [601487](http://omim.org/entry/601487) | NM_015869 | Familial Partial Lipodystrophy (FPLD3) and insulin resistance | [604367](http://omim.org/entry/604367) | Dominant | [Agarwal *et al* 2002 J Clin Endocrinol Metab 1: 408-411](http://www.ncbi.nlm.nih.gov/pubmed/?term=Agarwal+et+al+2002+J+Clin+Endocrinol+Metab+1%2C+408-411)  [Barroso *et al* 1999 Nature 402: 880-883](http://www.ncbi.nlm.nih.gov/pubmed/10622252) |
| *PPP1R15B*  [613257](https://www.omim.org/entry/613257) | NM_032833 | Juvenile-onset diabetes with microcephaly, epilepsy and intellectual disability | [616817](https://www.omim.org/entry/616817) | Recessive | [Abdulkarim *et al* 2015 Diabetes 64: 3951-3962](https://www.ncbi.nlm.nih.gov/pubmed/26159176) |
| *PTF1A*  [607194](http://omim.org/entry/607194) | NM_178161 | Permanent neonatal diabetes with cerebellar and pancreatic agenesis | [609069](http://omim.org/entry/609069) | Recessive | [Sellick *et al* 2004 Nat Genet 36: 1301-1305](http://www.ncbi.nlm.nih.gov/pubmed/?term=Sellcik+Nature+Genet+2004) |
| *RFX6*  [612659](http://omim.org/entry/612659) | NM_173560 | Permanent neonatal diabetes with pancreatic hypoplasia, intestinal atresia, and gallbladder aplasia or hypoplasia | [615710](https://omim.org/entry/615710) | Recessive | [Smith *et al* 2010 Nature 463: 775-780](http://www.ncbi.nlm.nih.gov/pubmed/?term=Smith+Nature+2010+RFX6) |
|  |  | MODY |  | Dominant | [Patel *et al* 2017 Nat Commun 8: 888](https://www.ncbi.nlm.nih.gov/pmc/articles/PMC5638866/) |
| *SIRT1*  [604479](https://www.omim.org/entry/604479) | NM_012238 | Monogenic autoimmune diabetes | Not assigned | Dominant | [Biason-Lauber *et al* 2013 Cell Metab. 17: 448-455](https://www.ncbi.nlm.nih.gov/pubmed/23473037) |
| *SLC2A2*  [138160](http://omim.org/entry/138160) | NM_000340 | Fanconi-Bickel syndrome | [227810](http://omim.org/entry/227810) | Recessive | [Santer *et al* 1997 Nat Genet 17: 324-326](http://www.ncbi.nlm.nih.gov/pubmed/?term=Santer+1997+Nature+Genetics) |
| *SLC19A2*  [603941](http://omim.org/entry/603941) | NM_006996 | Thiamine responsive megaloblastic anaemia, diabetes and deafness (TRMA) syndrome | [249270](http://omim.org/entry/249270) | Recessive | [Labay *et al* 1999 Nat Genet 22: 300-304](http://www.ncbi.nlm.nih.gov/pubmed/?term=Labay+Nature+Genet+1999) |
| *SLC29A3*  [612373](https://www.omim.org/entry/612373) | NM_018344 | H syndrome & PHID syndrome | [602782](https://www.omim.org/entry/602782) | Recessive | [Cliffe *et al* 2009 Hum Molec Genet 18: 2257-2265](https://www.ncbi.nlm.nih.gov/pubmed/19336477)  [Molho-Pessach *et al* 2008 Am J Hum Genet 83: 529-534](https://www.ncbi.nlm.nih.gov/pubmed/18940313) |
| *STAT1*  [600555](https://www.omim.org/entry/600555) | NM_007315 | Immunodeficiency 31C and IPEX-like phenotype | [614162](https://www.omim.org/entry/614162) | Dominant | [Uzel *et al* 2013 J Allergy Clin Immun 131: 1611-1623](https://www.ncbi.nlm.nih.gov/pubmed/23534974) |
| *STAT3*  [102582](http://omim.org/entry/102582) | NM_139276 | Neonatal diabetes and poly-autoimmune disease | [615952](http://omim.org/entry/615952) | Dominant | [Flanagan *et al* 2014 Nat Genet 46: 812-814](http://www.ncbi.nlm.nih.gov/pubmed/25038750) |
| *STAT5B*  [604260](https://www.omim.org/entry/604260) | NM_012448 | Growth hormone insensitivity with immunodeficiency | [245590](https://www.omim.org/entry/245590) | Recessive | [Kofoed *et al* 2003 N Engl J Med 349: 1139-147](https://www.ncbi.nlm.nih.gov/pubmed/13679528) |
| *TNFAIP3*  [191163](https://www.omim.org/entry/191163) | NM_001270508 | Familial Behcet-Like Auto-inflammatory Syndrome and autoimmune diabetes | [616744](https://www.omim.org/entry/616744) | Dominant | [Zhou *et al* 2016 Nat Genet 48: 67-73](https://www.ncbi.nlm.nih.gov/pubmed/26642243) |
| *TRMT10A*  [616013](http://www.omim.org/entry/616013) | NM_001134665 | Juvenile-onset diabetes with microcephaly, epilepsy and intellectual disability | [616033](http://www.omim.org/entry/616033#2) | Recessive | [Igoillo-Esteve *et al* 2013 PLoS Genet 9: e1003888](http://www.ncbi.nlm.nih.gov/pubmed/24204302) |
| *WFS1*  [606201](http://omim.org/entry/606201) | NM_006005 | Wolfram syndrome (Diabetes insipidus, diabetes mellitus, optic atrophy and deafness, DIDMOAD) | [222300](http://omim.org/entry/222300) | Recessive | [Inoue *et al* 1998 Nat Genet 20: 143-148](http://www.ncbi.nlm.nih.gov/pubmed/?term=Inoue+et+al+1998+Nat+Genet+20%2C+143-148)  [Strom *et al* 1998 Hum Mol Genet 7: 2021-2028](http://www.ncbi.nlm.nih.gov/pubmed/9817917) |
| *ZBTB20*  [606025](https://www.omim.org/entry/606025) | NM_001164342 | Primrose syndrome | [259050](https://www.omim.org/entry/259050) | Dominant  (*de novo*) | [Cordeddu *et al* 2014 Nat Genet 46: 815-817](https://www.ncbi.nlm.nih.gov/pubmed/25017102) |
| *ZFP57*  [612192](http://omim.org/entry/612192) | NM_001109809 | Transient neonatal diabetes | [601410](http://omim.org/entry/601410) | Recessive | [Mackay *et al* 2008 Nat Genet 40: 949-951](http://www.ncbi.nlm.nih.gov/pubmed/18622393) |

**Supplementary Table 2**. Type 1 diabetes SNPs included in the genetic risk score with weights. Effect allele is the risk increasing allele on the positive strand.

| SNP | Gene | Odds Ratio | Weight | Effect allele |
| --- | --- | --- | --- | --- |
| rs2187668, rs7454108 | *DR3/DR4-DQ8* | 48.18 | 3.87 |  |
|  | *DR3/DR3* | 21.12 | 3.05 |  |
|  | *DR4-DQ8/DR4-DQ8* | 21.98 | 3.09 |  |
|  | *DR4-DQ8/X* | 7.03 | 1.95 |  |
|  | *DR3/X* | 4.53 | 1.51 |  |
| rs1264813 | *HLA_A_24* | 1.54 | 0.43 | T |
| rs3129889 | *HLA_DRB1_15* | 14.88 | 2.7 | A |
| rs2476601 | *PTPN22* | 1.96 | 0.67 | A |
| rs689 | *INS* | 1.75 | 0.56 | T |
| rs12722495 | *IL2RA* | 1.58 | 0.46 | T |
| rs2292239 | *ERBB3* | 1.35 | 0.3 | T |
| rs10509540 | *C10orf59* | 1.33 | 0.29 | T |
